# Mimotopes selected with neutralizing antibodies against multiple subtypes of influenza A

**DOI:** 10.1186/1743-422X-8-542

**Published:** 2011-12-15

**Authors:** Yanwei Zhong, Jiong Cai, Chuanfu Zhang, Xiaoyan Xing, Enqiang Qin, Jing He, Panyong Mao, Jun Cheng, Kun Liu, Dongping Xu, Hongbin Song

**Affiliations:** 1 Pediatric Liver Disease Research Laboratory, Institute of Infectious Diseases, Beijing 302 Hospital, Beijing, China; 2 Department of Nuclear Medicine, PUMC Hospital, PUMC & CAMS, Beijing, China; 3 Institute of Disease Control and Prevention, Academy of Military Medical Sciences, Beijing, China; 4 Liver Center, Beijing Ditan Hospital, Capital Medical University, Beijing, China

**Keywords:** Influenza, Mimotopes, Phage display, Vaccination, Virus challenge

## Abstract

**Background:**

The mimotopes of viruses are considered as the good targets for vaccine design. We prepared mimotopes against multiple subtypes of influenza A and evaluate their immune responses in flu virus challenged Balb/c mice.

**Methods:**

The mimotopes of influenza A including pandemic H1N1, H3N2, H2N2 and H1N1 swine-origin influenza virus were screened by peptide phage display libraries, respectively. These mimotopes were engineered in one protein as multi- epitopes in Escherichia coli (E. coli) and purified. Balb/c mice were immunized using the multi-mimotopes protein and specific antibody responses were analyzed using hemagglutination inhibition (HI) assay and enzyme-linked immunosorbent assay (ELISA). The lung inflammation level was evaluated by hematoxylin and eosin (HE).

**Results:**

Linear heptopeptide and dodecapeptide mimotopes were obtained for these influenza virus. The recombinant multi-mimotopes protein was a 73 kDa fusion protein. Comparing immunized infected groups with unimmunized infected subsets, significant differences were observed in the body weight loss and survival rate. The antiserum contained higher HI Ab titer against H1N1 virus and the lung inflammation level were significantly decreased in immunized infected groups.

**Conclusions:**

Phage-displayed mimotopes against multiple subtypes of influenza A were accessible to the mouse immune system and triggered a humoral response to above virus.

## Background

Influenza A can cause significant morbidity and mortality levels in human. The human influenza A pandemics killed about millions of people worldwide over the past (1918 H1N1 Spanish, 1957 H2H2 Asian, 1968 H3N2 Hong Kong, and 2009 H1N1 Mexico) and seasonal influenza A killed more than 250,000 each year [1-3]. The pathogenic viruses are classified by their surface proteins: hemagglutinin and neuraminidase [4,5]. There are 16 hemagglutinin subtypes (H1-16) and 9 neuraminidase subtypes (N1-9) on the influenza viral surface [6]. Although Neuraminidase inhibitors and amantadine have been used to treat influenza patients, they have limited efficacy and their widespread use is likely to result in resistant viruses [7,8]. Consequently, vaccination remains the most effective strategy to prevent influenza virus attack [9,10]. Developing a new vaccine which induces a broad immune response against multiple subtypes of influenza A is a urgent strategy for the disease control.

The viruses mimotopes are considered to be good targets for the vaccine design since they can induce antibodies against both viral original and mutant antigen [11]. Protective immune responses by mimotope immunization have been verified in many infectious diseases [11-14]. The phage display libraries have been used for novel therapeutic and diagnostic drugs development in our and others previous studies [15-18]. Random peptide phage libraries provide rich resources for selecting sequences that mimic conformational epitopes (mimotopes) either structurally or immunologically [11]. The aim of this study was to prepare mimotopes against multiple subtypes of influenza A and evaluate its immune responses in Balb/c mice with flu virus challenge.

## Methods

### Antibodies

C179 monoclonal antibody (A/H2N2 subtype) was purchased from Takara Bio Inc (Otsu, Shiga, Japan); Mouse monoclonal antibody (IV.C102) against influenza virus A strain H1N1 was from Santa Cruz (Santa Cruz, CA, USA); Purified H3N2 goat polyclonal IgG specific to influenza A/Texas 1/77 was from Virostat (Portland, ME, USA); SIV sera were prepared from patients hospitalized by swine-origin influenza virus A/2009 and their binding activities were tested by ELISA. Endotoxin was removed by purification with polymyxin B chromatography. Endotoxin levels were < 0.1 unit/μg of protein by the Limulus Amebocyte Lysate QCL-1000 pyrogen test (Cambrex).

### Phage display libraries

Ph.D.-7, Ph.D.-12 and Ph.D.-C7C were produced by New England Biolabs, Inc (Ipswich, MA, USA), with random linear 7-mer, 12-mer or cyclic 7-mer peptides fused to minor coat proteins (pIII) of M13 filamentous phages.

### Screening of phage libraries for H2N2 antibody-reactive phages

C179 (0.2 ml, 10 μg/ml) was coated on three wells of 24-wells microplate at 4°C overnight. The coated wells were blocked with 2% bovine serum albumin (BSA) at 37°C for 1 h, then washed with Tris•HCl buffer solution (TBS) containing 0.1% Tween-20 (TBST) for 6 times. Ten microliter Ph.D.-7 (2 × 10^11 ^pfu), Ph.D.-12 (1.5 × 10^11 ^pfu) and Ph.D.-C7C (2 × 10^11 ^pfu) libraries diluted with 0.2 ml TBST were dropped into the coated wells respectively. The incubation wells were rocked gently for 30 min, followed by discarding nonbinding phages. The binding phages were eluted with 0.2 M Glycine-HCl (pH 2.2), 1 mg/ml BSA and neutralized with 1 M Tris•HCl (pH 9.1). The eluted phages were used to infect log-phase bacteria ER2738, concentrated by PEG precipitation and submitted to the second round of selection. The following selections were performed as above except that the Tween-20 concentration was raised from 0.1% to 0.5% in the wash steps. After 3 rounds of selection, the eluted phages were used for plaque isolation. 32 plaque clones were amplified for ELISA test and single-strand DNA preparation.

### Screening of phage libraries for H1N1 antibody-reactive phages

IV.C102 (0.2 ml, 10 μg/ml) was coated on three wells of 24-wells microplate at 4°C overnight. The blocking and bio-spanning procedures were carried as did in C179 antibody, except that the binding phages were eluted with IV.C102 (0.2 ml, 10 μg/ml). After 3 rounds of selection, the eluted phages were used for plaque isolation.

### Screening of phage libraries for H3N2 antibody-reactive phages

H3N2 polyclonal antibody (30 μg/ml) and goat IgG (100 μg/ml) were coated respectively. After blocking with BSA and washing with TBST for 6 times, Ph.D.-7, Ph.D.-12 and Ph.D.-C7C libraries were added into the goat IgG-coated wells for 30 min incubation. Then, the nonbinding phages were transferred to H3N2 polyclonal antibody-coated wells for additional 30 min incubation. After that, the non-binding phages were discarded, and the binding phages were eluted with H3N2 polyclonal antibody. The eluted phages were amplified in bacteria ER2738 and used in the following selections as above.

### Screening of phage libraries for swine-origin influenza antibody-reactive phages

Goat anti-human IgG (100 μg/ml) was coated on six wells. After blocking, three wells were added with diluted SIV sera (1:20), the others were added with human IgG (100 μg/ml). Then they were placed at 4°C overnight and washed with TBST for 6 times. Peptide phage display libraries were added into the IgG wells for 30 min incubation. Subsequently, the nonbinding phages solutions were transferred to the sera wells for 30 min incubation. Then, the binding phages at the sera wells were eluted with goat anti-human IgG. The eluted phages were used for plaque isolation at the end of the 3rd selection.

### Binding specificity of the selected phage by ELISA and DNA sequencing

Ninety-six-well plates were coated with mAb and BSA (10 μg/ml, 100 μl) by incubation at 4°C overnight, and blocked with 5% BSA in TBS. Affinity-selected phage were added to the wells and allowed to bind at 37°C for 1 h. After the unbounded phages were removed with 5% TBST, the bound phages were detected by incubation with peroxidase-labelled murine anti-M13 antibodies (Pharmacia). The bound peroxidase was determined by incubation with Opheny lenediamine dihydrochloride (Pierce Chemicals) in buffer (30 mM citrate, 70 mM Na2HPO4, and 0.02% H2O2, pH 5.5). When the reaction was stopped by the addition of 3 N HCl, A450 nm was determined with an ELISA reader (BioRad). All the assays were carried out in triplicate.

The phage from the 3rd biopanning eluate was cloned for immune-analysis. The nucleotide sequence of the gene III insert was determined as the instruction manual. The amino acid sequence of the insert was deduced from the nucleotide sequence and was compared with native influenza A. The sequences that appeared > 3 times among the selected phage clones were classified as the consensus sequence. The aligned amino acid sequences shared by three or more identical amino acids within the dodecapeptides (heptapeptides) were determined as the mimotopes of the matched protein sequences.

### Multi-mimotope gene synthesis

The 7- and 12-mer mimotopes of H1N1, H2N2, H3N2 and SIV were linked by GSGGS with the mimotope sequences of SIV7-SIV12-H1N17-H1N112-H3N27- H3N212-H2N27-H2N212. Each mimotope represents the peptide with the highest frequency on phage surface. The codon usage was optimized by species preference and GC content. The gene was synthesized with *Eco*R I/*Bam*H I enzyme site by Sangon (Shanghai, China).

### Multi-mimotope expression

The multi-mimotope gene was cut with *Eco*R I and *Bam*H I endonucleases, and ligated separately into precut pGEX-2 T-1, pGEX-4 T-1 and pET43a (+) plasmids. The ligation products were used to transform competent Trans 109 *E. coli *cells, which were selected on LB-AMP agar plates at 37°C for 12 h. Three AMP-resisitant clones were picked randomly for plasmids extraction, *Eco*R I/*Bam*H I digestion and gene sequencing. The confirmed plasmids with correct insert were transformed into competent BL21 (DE3) *E. coli *cells for protein expression. Four transformed bacteria BL21 (DE3) clones were picked from LB-AMP agar plates for culturing overnight in LB-AMP medium. The resultant bacteria were inoculated into 5 ml fresh medium, cultured to mid-log growth phase for protein expression induction with 1 mM IPTG. After 12 h induction, the bacteria sample was aliquot for SDS-PAGE analysis.

### Multi-mimotope purification

One hundred milliliters of BL21 (DE3) *E. coli *cells containing pET43a (+)-multi-mimotope plasmids were induced with IPTG for 12 h. The resultant medium was centrifuged with 8000× g for 10 min and the pellet was resuspended into 10 ml of 20 mM TBS (pH 7.9). The cells were broken by ultrasounding with 100 W for 100 s, followed by centrifuging to remove the cell debris. The supernatant was filtered through 0.22 μm membrane and then loaded onto pre-equilibrated Ni^2+^-NTA-resin. Then, the resin was rinsed by TBS containing 5 mM imidazole; the binding protein was eluted by TBS containing variable imidazole.

### *In vitro *binding

The recombinant multi-mimotope was coated on 96-well microplate with concentration of 10 μg/ml at 4°C overnight, followed by blocking with 2% BSA at 37°C for 2 h. The bait antibodies including C179, H1N1 monoclonal antibody, H3N2 polyclonal antibody and SIV sera were added into wells separately to incubate with coated protein at 37°C for 2 h. The wells were washed with PBST for 6 times. Then, HRP-conjugated secondary antibodies were added for binding at 37°C for 2 h. The TMB solution was then added into the wells for color development, which was stopped with 3 N HCl. The unrelated protein BSA was coated as control protein to determine the binding specificity of multi-mimotope to the antibodies.

### Animal immunisation

To evaluate the potential of the selected mimotopes as experimental vaccine candidates, purified phage mimotopes were used to immunise female inbred specific-pathogen-free BALB/c mice through intraperitoneal administration.

The multi-mimotope protein was concentrated to 1 mg/ml and injected intraperitoneally (50 μg, 100 μg, 200 μg per mouse) or subcutaneously (100 μg per mouse) into BALB/c mice (9 per group) as emulsion (1:1) with complete Freund's adjuvant (CFA) for the first immunization and with incomplete Freund's adjuvant (IFA) for the booster injection at 14 days later. The control group were injected with PBS. Ten days after the booster injection, except for control group, other 5 groups were challenged with 2 × 10^3 ^f.f.u A/Puerto Rico/8/1934 (H1N1) by intranasal inoculation of 50 μl per mouse. Mice were weighed on the day of virus challenge and then every three days for two weeks. Two weeks after the challenge, lungs were removed for pathological examination. Blood samples were taken to measure serum Ab titers by ELISA. Animals were conducted and approved by the Institutional Animal Care and Use Committee of the Academy of Military Medical Science, under protocol number 0054921. All experiments were performed according to institutional guidelines.

### Serum Ab assay by ELISA

The concentrations of IgG Abs against H1N1 influenza virus were measured by ELISA. Purified antigen was coated on the microtitre plates (100 μl/well, 5 μg/ml in coating solution, 0.1 M sodium bicarbonate, pH 9.6) (Corning, Corning, NY, USA) and incubated at 4°C overnight. Serial 2-fold dilutions of sera (100 μl/well) from each group of unimmunized or immunized and immunized infected were incubated for 1 h at 37°C. Goat anti-mouse IgG HRP (1:10,000 dilution with washing buffer) was used to detect IgG Abs and O-phenylenediamine dihydrochloride (Pierce Chemicals) was used as substrate for HRP and the reaction was monitored at an absorption of 492 nm using an ELISA reader (Labsystems Multiskan, Finland).

### The lung tissue pathological examination

Lung tissue samples were fixed in 10% formalin and embedded with paraffin,sections were cut at 5 μm thickness and were stained with hematoxylin eosin (HE).

### Statistical analysis

The data from test groups were evaluated by Student's*t*-test. The survival rates of mice in test and control groups were compared by using Fisher's exact test. All differences were considered significant at *P *values0.05.

## Results

### Screening, ELISA and sequences of different antibody-reactive phages

**Figure 1:**
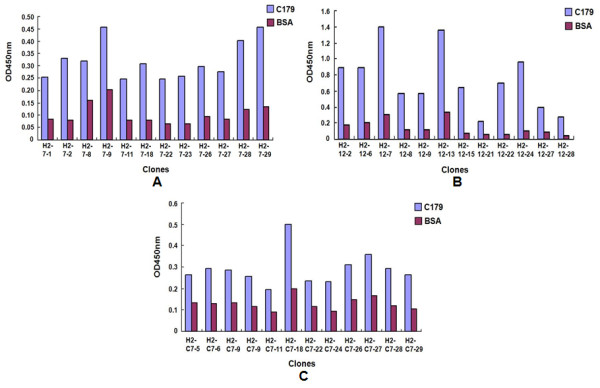
**The ELISA results of top 12 phage clones with higher C179/BSA ELISA signal ratio from Ph.D. -7, Ph.D. -12 and Ph.D. -C7C peptide phage-display libraries**. **a**: Phage clones from Ph.D. -7 peptide phage-display library. **b**: Phage clones from Ph.D. -12 peptide phage-display library; **c**: Phage clones from Ph.D. -C7C peptide phage-display library.

With H2N2 monoclonal antibody C179 as bait protein, binding phages from Ph.D.-7, Ph.D.-12 and Ph.D.-C7C peptide phage-display libraries were enriched by three rounds of binding-elution-amplification. Thirty-two binding phage clones were picked up randomly from every library for ELISA testing with C179 coated and uncoated wells (BSA blocked). Twelve phage clones with higher ELISA signal ratio (C179 to BSA) were chosen for single-strand DNA preparation and DNA sequencing. As shown in Figure 1, the overall binding affinity of linear 7-mer and cyclic 7-mer peptide-displayed phages to C179 was much lower than that of linear 12-mer group. The clone H2-12-22 had the highest ELISA signal ratio in linear 12-mer group, which was above 10. In linear 7-mer group, the clone H2-7-18 had the highest ELISA signal ratio of 3.89. In cyclic 7-mer group, the clone H2-C7-29 had the highest ELISA signal ratio of 2.55 (Figure 1). The selected phages were amplified in bacteria ER2738 and their single-strand DNA was extracted for gene sequencing. The peptide sequences were summarized in Table 1. The dominant sequences were considered as the mimotopes of the influenza A virus A/Okuda/57 strain, according to C179 monoclonal antibody. The linear heptopeptide WHWRLPS, linear dodecapeptide WHTHKWSLSAKA and cyclic cysteine-restricted heptopeptide NLSSSWI had low similarity (Table 1).

**Table 1:** The phage-displayed peptides bound to C179 monoclonal antibody and their frequencies

Linear 7-mer		Linear 12-mer		Cyclic 7-mer	
**Peptide sequences**	**Frequency**	**Peptide sequences**	**Frequency**	**Peptide sequences**	**Frequency**

WHWRLPS	10/12	WHTHKWSLSAKA	4/12	NLSSSWI	9/12

LHHKTHH	2/12	HHWKFFFSHPGA	2/12	NSGMFVR	3/12

		HHWKFFFSHPGE	2/12		

		LPFHGHKKPVLS	1/12		

		WPWWPGHTHRTI	1/12		

		HPMKQYRWRPSI	1/12		

		SPNYWFNKIHQH	1/12		

The dominant sequence displayed on linear heptopeptide phages binding to IV.C102 H1N1 monoclonal antibody was QWTWTQY, whereas the linear dodecapeptide was DCWQMDRKTCPL, cyclic heptopeptide was PLHARLP. All sequences and their frequencies were listed in Table 2.

**Table 2:** The phage-displayed peptides bound to IV.C102 monoclonal antibody and their frequencies

Linear 7-mer		Linear 12-mer		Cyclic 7-mer	
**Peptide sequences**	**Frequency**	**Peptide sequences**	**Frequency**	**Peptide sequences**	**Frequency**

QWTWTQY	4/12	DCWQMDRKTCPL	6/12	PLHARLP	10/12

DTLPLFI	1/12	NTPAWLNHTTVI	3/12	SLASLPA	2/12

MSLQQEH	1/12	LPAFFVTNQTQD	1/12		

ANTTPRH	1/12	TVHWWZTHGPLS	1/12		

MDAHHAL	1/12	SAIPTTWNPLAV	1/12		

ITAPHPH	1/12				

QRNQTQD	1/12				

QWNRTQY	1/12				

NTAPHPH	1/12				

The dominant sequence displayed on linear heptopeptide phages binding to swine-origin influenza virus A sera was ETKAWWL, whereas the linear dodecapeptide was QAHNWYNHKPLP, cyclic heptopeptide was PLHARLP. All sequences and their frequencies were listed in Table 3.

**Table 3:** The phage-displayed peptides bound to SIV sera and their frequencies

Linear 7-mer		Linear 12-mer		Cyclic 7-mer	
**Peptide sequences**	**Frequency**	**Peptide sequences**	**Frequency**	**Peptide sequences**	**Frequency**

ETKAWWL	7/12	QAHNWYNHKPLP	6/12	PLHARLP	6/12

LASKPMP	4/12	VHNNAARTGSPP	2/12	RHLPLTP	1/12

QAHTIST	1/12	VHNHANDPGSPP	1/12	SLPLTGQ	1/12

		QELYPYSPHIHV	1/12	PSYPLSF	1/12

		FSHELSWKPRKA	1/12	RDISPLA	1/12

		AHTHSKERVQTI	1/12	YGWPIYS	1/12

				NLSSSWT	1/12

The dominant sequence displayed on linear heptopeptide phages binding to influenza A virus A/H3N2 polyclonal IgG was WPWHNHR, whereas the linear dodecapeptide was VWSTPPHADGPA, cyclic heptopeptide was LGALSHT. All sequences and their frequencies were listed in Table 4.

**Table 4:** The phage-displayed peptides bound to H3N2 polyclonal antibody and their frequencies

Linear 7-mer		Linear 12-mer		Cyclic 7-mer	
**Peptide sequences**	**Frequency**	**Peptide sequences**	**Frequency**	**Peptide sequences**	**Frequency**

WPWHNHR	6/12	VWSTPPHADGPA	4/12	LGALSHT	8/12

ASINSSL	2/12	HAPWRHHQASPK	3/12	SPVLPFL	1/12

QSERAIQ	1/12	FPAHPAWTIGSM	1/12	THEPSGR	1/12

TSLPTIV	1/12	YTPLSSASPWGP	1/12	SAPRQAD	1/12

AFSYHIH	1/12	GMSLLHGQRPHT	1/12	SLPLTGQ	1/12

NMWQALN	1/12	VSRHQSWHPHDL	1/12		

		EREAHQLHSHHK	1/12		

### Multi-mimotope gene synthesis

The multi-mimotope SIV7-SIV12-H1N17-H1N112-H3N27-H3N212-H2N27- H2N212 of influenza A covered the mimotopes of H2N2, H1N1, H3N2 and SIV subtypes. Each subtype contained heptopeptide and dodecapeptide mimotopes. The whole amino acids sequence and nucleotide sequence were shown in Table 5

**Table 5:** The amino acids sequence and nucleotide sequence of multi-mimotope of influenza A

E T K A W W L G S G G S Q A H N W Y N H K P L P G S G
gaaactaaagcatggtggctgggttctggtggttctcaggctcataactggtataaccataagccactgccaggttccggt

G S Q W T W T Q Y G S G G S D C W Q M D R K T C P L G

ggttctcagtggacttggacgcagtacggtagcggtggctccgactgttggcagatggatcgcaaaacctgtccactgggt

S G G S W P W H N H R G S G G S V W S T P P H A D G P

tctggcggtagctggccttggcataaccatcgtggcagcggtggttctgtttggtctactccaccgcatgctgatggtcca

A G S G G S W H W R L P S G S G G S W H T H K W S L S

gctggctctggcggttcttggcattggcgtctgccatctggctctggtggttcttggcacactcacaaatggtctctgtct

A K A

gctaaagca

### Multi-mimotope expression and purification

The multi-mimotope gene was subcloned into pGEX-2 T-1, pGEX-4 T-1 and pET43a (+) expression plasmids, respectively. Their reading frames were confirmed by *EcoR *I/*BamH *I digestion and gene sequencing. After transforming into BL21 (DE3) *E. coli *cells and inducing with IPTG, the multi-mimotope genes were expressed as GST-fused protein with pGEX-2 T-1, pGEX-4 T-1 plasmids or as Nus/His6-fused protein with pET43a (+) plasmid. The proteins were of 42KD and 73KD in respective fused forms.

**Figure 2:**
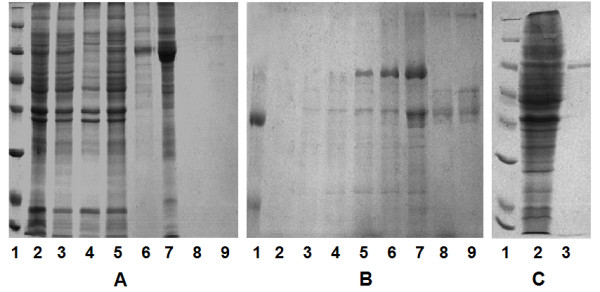
**The purification of recombinant multi-mimotope of influenza A virus**. **a**: The multi-mimotope was expressed in soluble form in bacteria and purified with affinity chromatography (lane 1: protein marker: 116.0, 66.2, 45.0, 35.0, 25.0, 18.4, 14.4KDa; lane 2: multi-mimotope gene was transferred to bacteria and induced with IPTG; lane 3: supernatant of ultrasound-broken bacteria; lane 4: pellete of ultrasound-broken bacteria; lane 5: flow-through of supernatant loaded on Ni^2+^-NTA-resin; lane 6: first 0.25 ml elution from 0.25 ml Ni^2+^-NTA-resin with 60 mM imidazole; lane 7: second 0.25 ml elution from Ni^2+^-NTA-resin with 60 mM imidazole; lane 8-9: elution from Ni^2+^-NTA-resin with 1 M imidazole). **b**: The optimized concentration gradient between 5 and 60 mM imidazole for affinity chromatography (lane 1: protein marker: 97.4, 66.4, 43.0KDa; lane 2-3: elution from Ni^2+^-NTA-resin with 5 mM imidazole; lane 4-5: elution from Ni^2+^-NTA-resin with 10 mM imidazole; lane 6-7: elution from Ni^2+^-NTA-resin with 30 mM imidazole; lane 8-9: elution from Ni^2+^-NTA-resin with 60 mM imidazole). **c**: The multi-mimotope was purified with repeat affinity chromatography (lane 1: protein marker: 116.0, 66.2, 45.0, 35.0, 25.0, 18.4, 14.4KDa; lane 2: supernatant of ultrasound-broken bacteria; lane 3: multi-mimotope purified by repeat affinity chromatography).

Large portion of Nus/His6-fusion multi-mimotope was produced as soluble form; in contrast, the GST-fusion proteins were expressed as insoluble form. So, BL21 (DE3) *E. coli *cells containing pET43a (+)-multi-mimotope plasmids were chosen for induction with IPTG. The Nus/His6-fusion multi-mimotope with endogenous His6 tag was bound by Ni^2+^-NTA-resin and eluted with TBS containing 60 mM imidazole (Figure 2a). The imidazole gradient was further investigated with the consequence that fusion protein could be eluted completely between 10 to 30 mM imidazole in TBS (Figure 2b). The eluted protein was dialyzed against TBS containing 5 mM imidazole and loaded on pre-equilibrated Ni^2+^-NTA-resin for further affinity purification. This time, the eluted protein was of high purity (Figure 2c).

### *In vitro *binding

**Figure 3:**
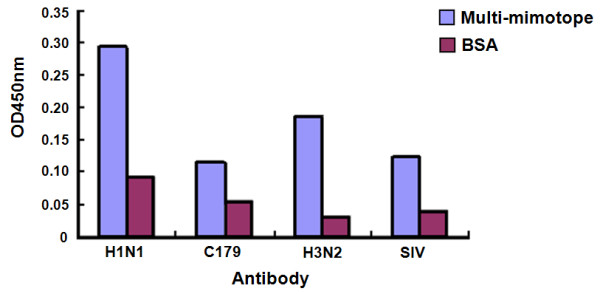
**The *in vitro *binding activities of recombinant multi-mimotope**.

The results (Figure 3) showed that the recombinant protein could be bound by H2N2 monoclonal antibody (C179), H1N1 monoclonal antibody (IV.C102), goat H3N2 polyclonal antibody and human SIV sera, respectively. But the control protein BSA could not.

### Prophylactic efficacy studies in mice

**Figure 4:**
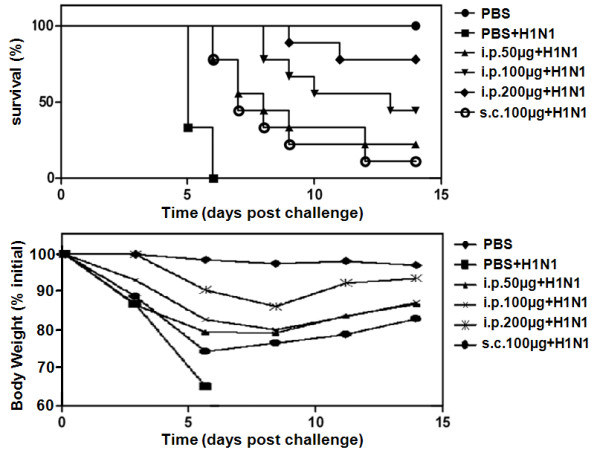
**The prophylactic effect of multi-mimotope agaist influenza virus challenge**. **a**: The survival rate of mice after virus challenge following multi-mimotope vaccination. **b**: The body weight loss of mice after virus challenge following multi-mimotope vaccination.

Fourteen days after A/Puerto Rico/8/1934 (H1N1) virus challenge, 9 of the 9 mice in PBS group, 7 of the 9 mice in 50 μg multi-mimotope i.p. group, 5 of the 9 mice in 100 μg multi-mimotope i.p. group and 8 of the 9 mice in 100 μg multi-mimotope s.c. group died. However, only 2 of the 9 mice in 200 μg multi-mimotope i.p. group died (Figure 4a). Six days after virus challenge, mice in PBS group lost 32% body weight, compared with 19% in 50 μg multi-mimotope i.p. group, 16% in 100 μg multi-mimotope i.p. group, 8% in 200 μg multi-mimotope i.p. group and 23% in 100 μg multi-mimotope s.c. group (Figure 4b). These findings suggested that multi-mimotope effectively reduced infection of influenza virus A.

**Figure 5:**
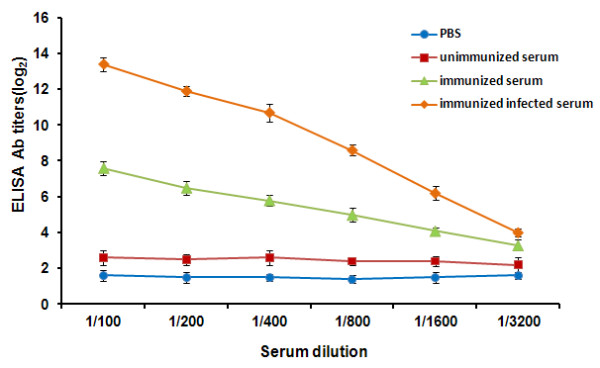
**Serum IgG Ab titers were detected by ELISA**. Nine mice were immunised with 200 μg multi-mimotope. The mice were bled once (unimmunized serum), and were bled every 10 days after the booster immunization (immunized serum). Ten days after the booster injection, mice were challenged with H1N1 virus by intranasal inoculation of 50 μl per mouse. Two weeks after challenge, mice were sacrificed for the anti-H1N1 IgG Ab detection by ELISA (immunized infected serum). Triplicate ELISAs were performed to test each serum sample. All assays were carried out in triplicate and the error bars indicate standard deviation.

Sera were collected and pooled from mice infected with A/PR8 (H1N1) influenza virus. The ELISA method was used to detect IgG Ab titers against H1N1 influenza viruses. As shown in Figure 5, the immune responses to PBS and unimmunized control mice were very low. For the mice, immunization with 200 μg multi-mimotope induced antigen-specific Abs. The specific IgG Abs in immunized mice after infected with H1N1 influenza virus were significantly increased.

### The lung tissue pathological changes

**Figure 6:**
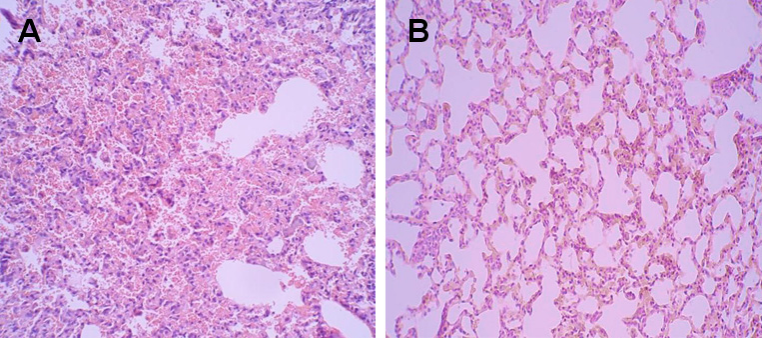
**The lung tissue pathological changes**. A: the unimmunized infected group (×200). The lung tissue pathological changes from the unimmunized infected groups died on day 6. Necrotizing interstitial pneumonia was found in all cases of death. The pulmonary tissue exhibited hyperemia,hemorrhage,and inflammatory exudation. B: the 200 μg multi-mimotope immunized infected group (×200). The lung tissue pathological changes from the 200 μg multi-mimotopes immunized infected group on day 14. Showing the lung inflammation level were significantly decreased compared with those of their matched unimmunized infected group.

Gross lesions were observed in the lungs of mice of the unimmunized infected groups,including pulmonary hyperemia,hemorrhage and consolidation. Importantly, the lung inflammation levels of the 200 μg multi-mimotope immunized infected group were significantly decreased compared with those of the matched unimmunized infected groups. Necrotizing interstitial pneumonia was found with light microscope in all cases of death in the unimmunized infected groups. The pulmonary tissue exhibited hyperemia, emorrhage and inflammatory exudation, leading to consolidation. The lumina of alveoli and bronchioles were variably filled with protein-rich edema fluid, fibrin, erythrocytes and cell debris, admixed with many neutrophils and lymphocytes in the unimmunized infected groups (Figure 6).

## Discussion

Current trivalent influenza vaccines can elicit production of neutralization antibody to benefit human beings [5,19-23]. However, the influenza vaccine must be updated each year based on global influenza surveillance due to rapid genetic shift and drift [9,10]. Developing a new vaccine that induces neutralization antibodies against multiple subtypes of influenza A is a promising strategy for the disease control [24]. It has been reported that mimotopes induce production of protective antibodies, and consequently, become candidates for the development of potential vaccines [25-28]. The phage-displayed mimotopes from random peptide libraries have recently been shown to be possible vaccine components that do not necessarily represent the structural equivalents of the original antigen, but provide functional images that could replace the original epitopes for vaccine development [29].

In the case of mimotope immunisation, several studies have shown effective responses *in vivo *[30,31]. Furthermore, protective immune responses by mimotope immunisation have been verified in many infectious diseases [11-14].

Monoclonal antibody C179, antigen binding fragment (Fab) CR6261 and single-chain variable fragment antibody (scFv) F10 recognize conserved epitope of hemagglutinin across different subtypes of influenza A viruses [1,3,15]. To mimic the conformational structure of the conserved motif, the peptide phage display technique was used in this paper to screen the mimotope with commercial C179 monoclonal antibody. Although C179 was produced by immunization of influenza A virus A/Okuda/57 strain (H2N2 subtype) can block membrane fusion rather than cell attachment and protect mice against viral challenge [32]. And its binding activities can be detected in H1 influenza A viruses, and possibly in H4 to H6, H8, H9, H11 to H14 and H16 influenza A viruses. In addition, H3N2 and H1N1 antibodies, especially swine-origin influenza sera were used to screen different types of mimotopes. The Ph.D.-7, Ph.D.-12 and Ph.D.-C7C peptide phage-display libraries with different lengths and formats of peptides were utilized to screen mimotopes. The individual mimotope was linked by GSGGS with the sequence SIV7-SIV12-H1N17-H1N112-H3N27-H3N212-H2N27-H2N212, which was used to test whether the synthetic gene with multiple GSGGS inserting affected the expression. The synthetic gene with multiple GSGGS was expressed successfully in *E. coli*, which was confirmed by SDS-PAGE [33].

In recent years, the universal influenza vaccines have been under investigation worldwide, including conserved epitope of surface M2 [34-36]. However all developed vaccines were far from the clinical needs [37]. In this paper, we utilized C179 to screen the mimotopes to hemagglutinin of influenza virus. The recombinant multi-mimotope covered the other mimotopes of hemagglutinin to increase its efficacy and versatility. The multi-mimotopes effectively protected animals from influenza A virus challenge. The recombinant multi-mimotopes could provide a novel and promising vaccine candidate for inducing a broad immune response.

## Competing interests

The authors declare that they have no competing interests.

## Authors' contributions

YWZ, DPX, HBS conceived the study and revised the manuscript critically for important intellectual content. YWZ, JC and CFZ made substantial contributions to its design, acquisition, analysis and interpretation of data. XYX, EQQ, JH performed experiments. PYM, JC, KL, SSZ participated in the design, analysis and interpretation of data. All authors read and approved the final manuscript.
